# A Case Report on Radiation-Induced Cerebral Vasculopathy in a Long-Term Survivor of Childhood Medulloblastoma

**DOI:** 10.7759/cureus.68213

**Published:** 2024-08-30

**Authors:** Sara C Esteves, Kathryn A Szymanski, Michael S Kuwabara

**Affiliations:** 1 Neuroradiology, Creighton University School of Medicine, Phoenix, USA; 2 Neuroradiology, Phoenix Children's Hospital, Phoenix, USA

**Keywords:** black blood sequencing, childhood radiotherapy, arterial spin-labeled imaging, metastatic medulloblastoma, cerebral irradiation, radiation-induced cerebral vasculopathy

## Abstract

This case report discusses a patient diagnosed with radiation-induced cerebral vasculopathy who presented after cerebral irradiation of metastatic medulloblastoma to raise awareness of radiation-induced cerebral vasculopathy. Because radiation therapy has revolutionized treatment for children with brain cancers, radiation-induced vasculopathy is becoming ever more prominent, and its recognition is crucial to implementing early treatment strategies to improve patient outcomes. Currently, medical management is poorly defined, largely unexamined, and poorly studied. Because the clinical features of this disease are nonspecific, radiation-induced cerebral vasculopathy remains a diagnosis of exclusion and an essential addition to the differential diagnosis. Discussion regarding standardized treatment, screening, and guidelines is necessary to improve treatment and survival.

## Introduction

Radiation therapy is a widely used cancer treatment for eradicating cancer cells [1] and improving overall survival [2]. While radiation therapy has revolutionized treatment for children with brain tumors, it is not devoid of significant late adverse effects, especially vasculopathy [2]. Radiation-induced cerebral vasculopathy presents in patients treated with cranial irradiation and can affect small and/or large arteries [3]. Radiation-induced vasculopathy is particularly prevalent among long-term survivors of childhood brain cancer [2]. Radiation contributes to vasculopathy by inducing the production of reactive oxygen species and pro-inflammatory/fibrogenic cytokines from direct endothelial injury and interfering with angiogenic pathways and vascular remodeling [2].

This case report was previously presented as a poster at the 2024 Creighton University Research Week on March 26, 2024.

## Case presentation

A 10-year-old male presented to an outside hospital with dizziness, lethargy, and fatigue. The patient's mother reported that the patient had episodes of intermittent dizziness over the previous month. Past medical history was significant for metastatic medulloblastoma at age five years, treated with chemotherapy without concurrent carboplatin, primary tumor resection, and radiation, in remission without treatment for four years, and resultant growth hormone deficiency and hypothyroidism. A head CT was completed at an outside hospital, and no acute intracranial abnormality was observed. Blood work completed here demonstrated mild normocytic anemia with hemoglobin of 11.4, but otherwise, complete blood count, complete metabolic panel, coagulation profile, and cortisol were unremarkable. The patient was transferred to our institution for further evaluation.

CT and MRI were obtained concurrently via stroke protocol. Initial CT head without contrast also showed postoperative changes consistent with resected posterior fossa tumor but did not demonstrate acute abnormalities. MRI re-demonstrated these findings and showed right internal carotid artery (ICA) stenosis and questionable new gliosis in the right basal ganglia. Imaging findings suggested focal cerebral arteriopathy versus radiation-induced vasculopathy, though the former was initially favored.

**Figure 1:**
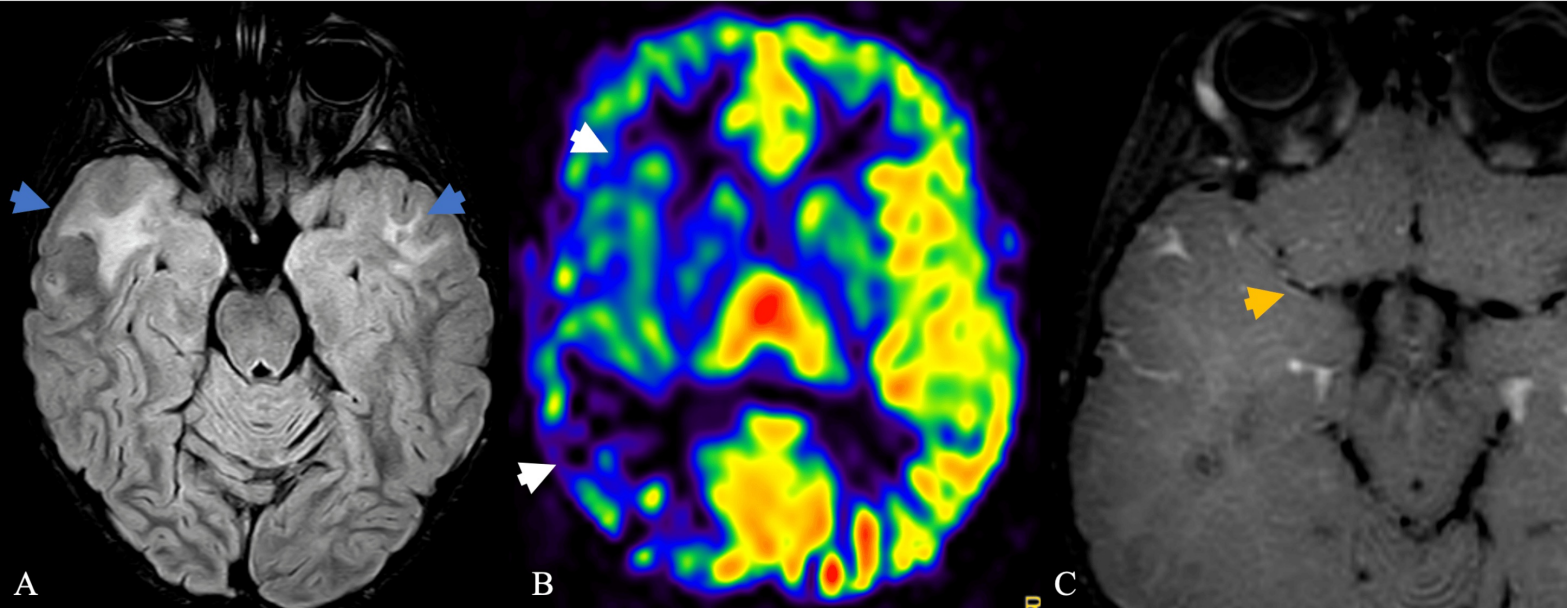
Axial MRI brain was acquired during hospitalization, approximately five years after the last brain radiation (A) FLAIR sequence shows anterior temporal lobe white matter hyperintensity (blue arrow). (B) Arterial spin-labeled imaging of the brain at the level of the caudate and putamen shows decreased blood flow within the right MCA territory (white arrow). (C) Post-contrast black blood imaging through the level of the circle of Willis shows mild enhancement circumferentially around the area of the narrowed M1 segment of the right MCA (yellow arrow). FLAIR: fluid-attenuated inversion recovery; MCA: middle cerebral artery

**Figure 2:**
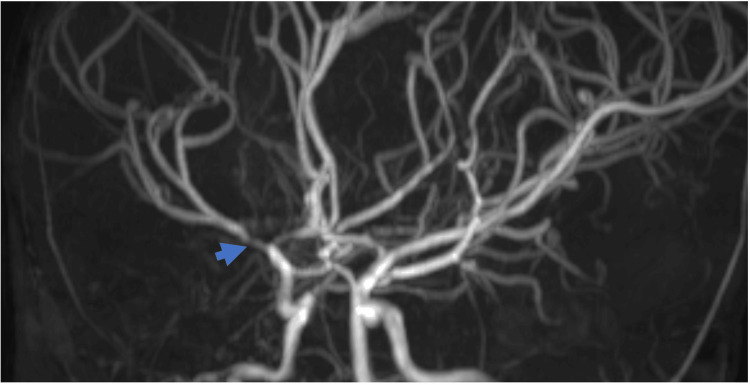
3D time of flight non-contrast MRA of the head acquired during admission demonstrating severe narrowing of the proximal right M1 segment of the right MCA (blue arrow) MRA: magnetic resonance angiography

At this point, neurology was consulted and recommended admission, steroids, and aspirin pending further exams and lumbar puncture to follow. Follow-up magnetic resonance imaging (MRI) of the brain and spine with and without contrast (Figure 1) and magnetic resonance angiography (MRA) head without contrast (Figure 2) were obtained the following day and confirmed the new area of gliosis in the right basal ganglia. Circumferential vessel wall enhancement (Figure 1C) with progressive narrowing of the right ICA terminus, M1 and A1 segments, and proximal segments of the right anterior cerebral artery (ACA) and middle cerebral artery (MCA) were present. Similar though more subtle changes were visible in the left ICA terminus and left proximal ACA and MCA segments. Multifocal susceptibility foci were also noted and were concerning for microhemorrhage versus post-radiation-induced cavernous malformations. There were no moyamoya collateral vessels, making a diagnosis of moyamoya disease unlikely. In addition, imaging and lab results did not suggest an acute infectious etiology.

**Figure 3:**
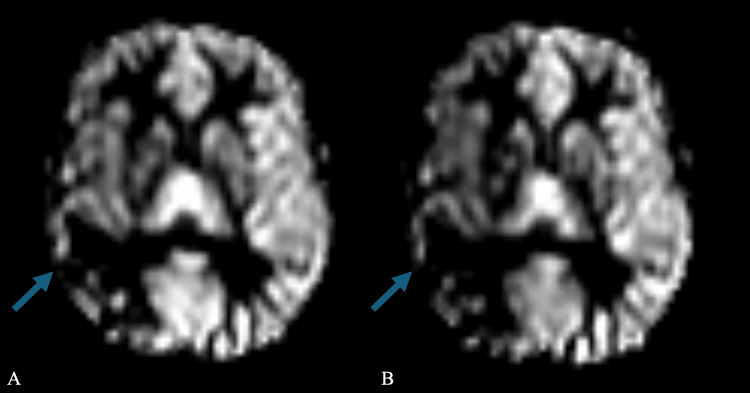
MRI pre-Diamox (A) showed decreased blood flow in the right middle cerebral artery territory, and post-Diamox (B) demonstrated further decreased flow in the same right middle cerebral artery territory, suggestive of maximal flow compensation with poor vascular reserve

**Figure 4:**
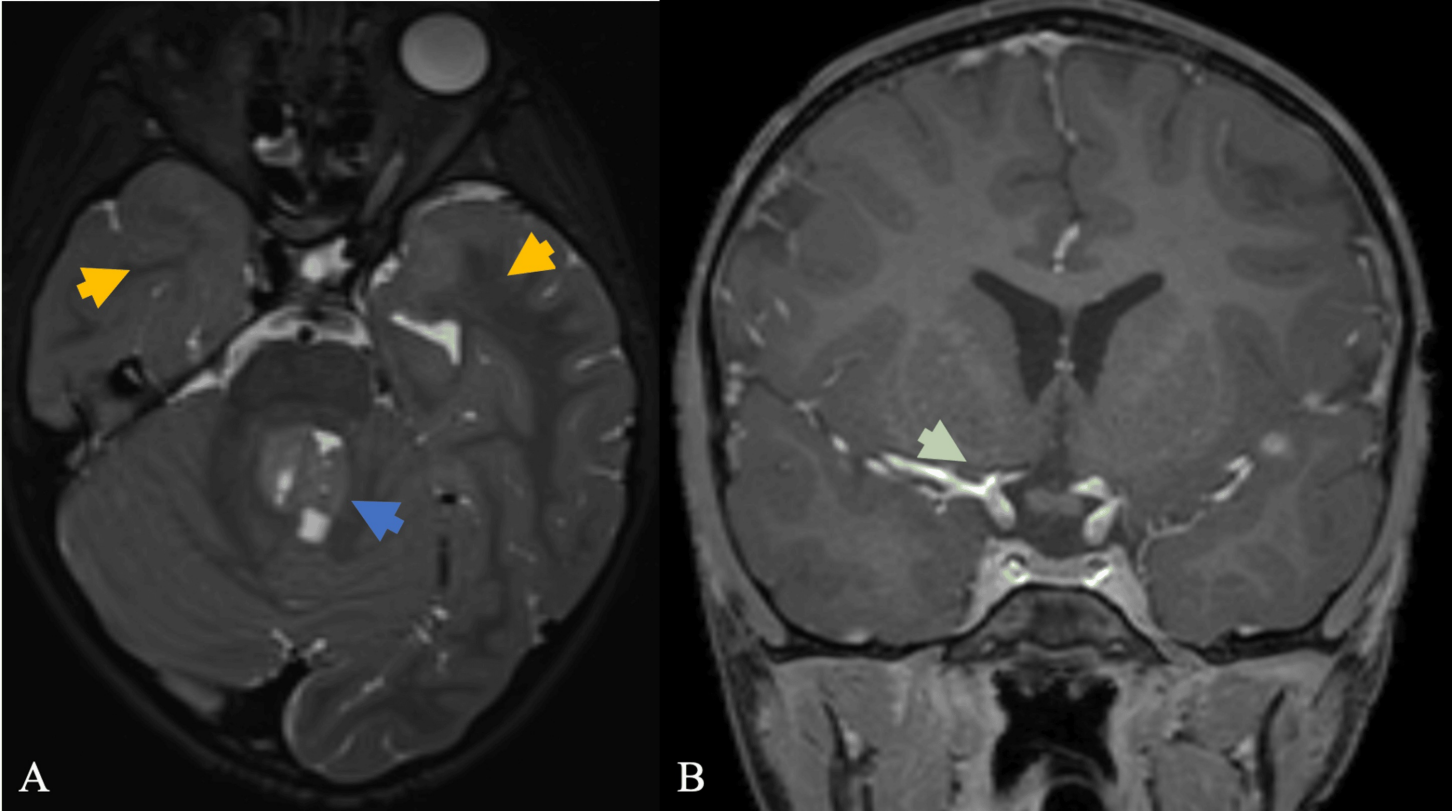
(A) Pre-radiation axial T2-weighted MRI through the skull base showing a T2 hyperintense mass in the fourth ventricle (blue arrow), and no abnormal signal is demonstrated in the anterior temporal lobe white matter (yellow arrow). (B) Immediate post-radiation coronal gadolinium-enhanced T1-weighted MRI through the anterior circulation showed a normal caliber M1 segment of the right MCA (green arrow) MRA: middle cerebral artery

The constellation of findings favored radiation-induced cerebral vasculopathy. MRI pre- and post-Diamox suggested maximal flow compensation, with the right side predominant decreased blood flow post-Diamox administration (Figure 3). Diamox should vasodilate blood vessels and increase blood flow; however, this paradoxical diminishing of blood flow, especially on the right, demonstrates preexisting maximal vasodilation, suggesting blood is being shunted elsewhere to more vasodilated blood vessels (Figure 3). Additionally, imaging demonstrated confluent areas of white matter hyperintense signal on (fluid-attenuated inversion recovery (FLAIR) within the anterior temporal lobes (Figure 1A). In comparison, studies performed one and two years prior include less conspicuous narrowing of aforementioned segments, and studies performed five years prior, before radiation, do not demonstrate narrowing (Figure 4), suggesting a progressive nature with collateralized flow. In the absence of alternative diagnoses, the patient was diagnosed with radiation-induced cerebral vasculopathy. 

Throughout the hospitalization, the patient had a headache and two episodes of emesis and was described by his mother to be intermittently off and with slower responsiveness. All the symptoms resolved, and the patient was discharged on prophylactic aspirin for outpatient follow-up with neurology and neurosurgery to evaluate for potential revascularizations and/or any other interventions.

The patient followed up with neurology several weeks later without any return of symptoms. He subsequently underwent an encephaloduroarteriosynangiosis after a couple of months and postoperatively reported several episodes of dizziness, one episode of left facial droop lasting several minutes, and intermittent tingling of his left first three fingers in the morning, which resolved spontaneously. While encephaloduroarteriosynangiosis provided a framework for angiogenesis, the patient remains at increased risk for stroke until angiogenesis occurs. Postoperatively, it was recommended that the patient lay flat, increase hydration, and avoid strenuous exercise to decrease dizziness events and the risk of stroke. Neurosurgery will follow up with imaging in the future.

## Discussion

Patients who undergo radiotherapy frequently experience toxicity-induced side effects, including vasculopathy. Smaller caliber vessels are particularly affected, and organs with high capillary concentration are notably susceptible [4]. Acute and chronic effects of radiation are triggered by endothelial cell apoptosis, senescence, and alterations in endothelial cell hemostasis, with signaling cascade disruption [4]. Disruption in signaling may affect the anti-inflammatory, antifibrinogenic, anticoagulant, antiatherogenic, and vasoactive abilities of cells [4].

This case report discusses a patient diagnosed with radiation-induced cerebral vasculopathy who presented four years after cerebral irradiation with an altered mental status and evidence of a previous stroke in the basal ganglia. The onset of radiation-induced vasculopathy can appear 4 months to 24 years following radiation therapy [3]. Clinical features are not pathognomonic for radiation-induced vasculopathy but are shared among cerebral infarction, transient ischemic attack, and moyamoya disease [3]. Radiation-induced vasculopathy remains a diagnosis of exclusion among patients with a history of radiation therapy to the afflicted vessels [3]. Another report remarked on a case of CNS radiation-induced vasculopathy 30 years following radiation exposure that could have been detected sooner through monitoring and detailed history-taking [3]. This case report included images of vessel narrowing affecting the MCA and ACA bilaterally and an old infarct similar to the presentation of this case [3]. Therefore, it is critical to consider radiation-induced vasculopathy among the differential diagnosis when risk factors are present.

The most critical risk factor for radiation-induced vasculopathy is a history of childhood radiotherapy; however, additional risk factors include adjunctive chemotherapy, a higher radiation dose, and other vascular risk factors [1]. Imaging findings on angiography, CTA, and MRA include diffuse steno-occlusions involving the common and internal carotid arteries [1]. Contrast-enhanced MR imaging utilizing black blood sequencing may also reveal arterial wall thickening and enhancement among radiation-induced large vessel vasculopathy, which may serve as a diagnostic clue when differentiating this disease from idiopathic moyamoya disease [5].

The treatment protocol is a patient-by-patient approach that may address cardiovascular risk factors like hypertension and diabetes, anticoagulation or anti-lipid medications, carotid endarterectomy, and carotid artery stenting [6]. Moreover, when possible, an emphasis should be placed on prevention. The therapeutic ratio, or the optimal balance between tumor control and normal tissue complications, must be considered when administering radiation [4]. Modern technological advances in radiation therapy strive to minimize radiation dose delivered to normal tissue and will hopefully reduce the incidence of radiation-induced vasculopathy [7]. Anti-vascular endothelial growth factor (VEGF) is a potential preventative treatment for radiation-induced cerebral necrosis with success in clinical practice [7]. It is proposed to act by disrupting VEGF upregulation, tissue edema, and hypoxia [7]. Additionally, genome-wide association studies have started to identify single nucleotide polymorphisms imputed to toxicity following radiation therapy [8]. In the future, this can be used to identify patients predisposed to radiation-induced side effects and alter the patient’s unique treatment plan.

This case was shared to raise awareness of radiation-induced cerebral vasculopathy as a complication of radiation therapy and to inform providers of symptoms and key imaging findings. Because of increased radiation approaches and patient survival, radiation-induced vasculopathy is becoming ever more prominent, and its recognition is crucial to implementing early treatment strategies [6]. Because medical management is poorly defined and remains poorly studied, we hope to inspire interest in creating standardized care and improving survival in radiation-induced vasculopathy [6].

## Conclusions

Radiation-induced cerebral vasculopathy is a long-term complication of radiotherapy, affecting individuals who have received radiation to the affected vessels. It is especially common among survivors of childhood brain cancers, as radiation therapy has revolutionized treatment for children with brain cancers. With improvements in survival of childhood cancers, it is crucial to include radiation-induced vasculopathy as an important differential diagnosis when treating brain cancer survivors presenting with suspicious neurologic symptoms. The threshold for brain imaging should be low, as the potential for radiation-induced vasculopathy is known, and early detection can aid in preventing progression. Further, survivors of childhood brain cancer require surveillance for long-term complications of radiotherapy. Preventative measures should be taken, and anti-VEGF should be discussed among patients and their providers.

Radiation-induced vasculopathy remains a poorly recognized condition, and so standardized treatment, screening, and guidelines are necessary to improve survival of this condition. We recommend future studies to follow patients who underwent radiotherapy for childhood brain cancer, monitor them yearly, and identify those who developed radiation-induced cerebral vasculopathy. Further studies could track patients who underwent childhood brain cancer, monitor them yearly, and identify those who developed radiation-induced cerebral vasculopathy. These studies could expand on understanding risk factors; however, the resources and time requirements would be a significant challenge. In addition, a Delphi study could be conducted to form a consensus from experts in the field on the treatment protocol to follow for this since it is a patient-by-patient approach.
